# Thin-Layer Polymer Wrapped Enzymes Encapsulated in Hierarchically Mesoporous Silica with High Activity and Enhanced Stability

**DOI:** 10.1038/srep04421

**Published:** 2014-03-21

**Authors:** Fang Zhang, Meitao Wang, Chao Liang, Huangyong Jiang, Jian Shen, Hexing Li

**Affiliations:** 1 The Education Ministry Key Lab of Resource Chemistry and Shanghai Key Laboratory of Rare Earth Functional Materials, Shanghai Normal University, Shanghai 200234, China

## Abstract

A novel soft-hard cooperative approach was developed to synthesize bioactive mesoporous composite by pre-wrapping Penicillin G amidase with poly(acrylaimde) nanogel skin and subsequently incorporating such Penicillin G amidase nanocapsules into hierarchically mesoporous silica. The as-received bioactive mesoporous composite exhibited comparable activity and extraordinarily high stability in comparison with native Penicillin G amidase and could be used repetitively in the water-medium hydrolysis of penicillin G potassium salt. Furthermore, this strategy could be extended to the synthesis of multifunctional bioactive mesoporous composite by simultaneously introducing glucose oxidase nanocapsules and horseradish peroxidase nanocapsules into hierarchically mesoporous silica, which demonstrated a synergic effect in one-pot tandem oxidation reaction. Improvements in the catalytic performances were attributed to the combinational unique structure from soft polymer skin and hard inorganic mesoporous silica shell, which cooperatively helped enzyme molecules to retain their appropriate geometry and simultaneously decreased the enzyme-support negative interaction and mass transfer limitation under heterogeneous conditions.

With the increasing concern about environmental pollution and energy crisis, green chemistry has received more and more attentions owing to the new concept of chemical synthesis with high efficiency (atomic economic) and low environmental pollution from raw materials, catalysts, solvents, reaction and isolation process, products and side-products etc^1^. Biocatalysis provides an ideal pathway to realize green chemistry due to the high activity and chemo-, regio-, and stereo-selectivity under mild reaction conditions^2^. However, most biocatalysts (enzymes) exhibit poor stability, which is quite sensitive toward reaction conditions. Meanwhile, enzymes are difficult to be separated from the reaction system, which limits their recycle and reuse, leading to high cost and the contamination of the products^3^,^4^. Immobilized enzymes could be easily recovered and also show enhanced stability^5^,^6^. Although excellent progress has been made in the preparation of a wide variety of immobilized enzymes, their industrial application is still limited by great decrease in activity comparing to the naked enzymes due to several inherent drawbacks from standpoint of catalysis. One is the change of enzyme conformation that is sensitive to the reactions^7^. Besides, the strong enzyme-support interaction and the enhanced diffusion limit are also harmful to the catalytic performances of immobilized enzymes^8^,^9^,^10^,^11^,^12^. In addition, the structure of the immobilized enzyme might be affected by the local surface geometry and surface chemistry of the nanoscale supports^13^. To date, the development of novel immobilized enzymes is still the major research topic in academy and industry.

Several different strategies including functionalizing supports surface^14^, incorporating biocompatible components inside the carriers^15^ and utilizing biomimetic materials^16^ as the supports have already been reported in the construction of a favourable solid matrix for the immobilized enzymes. However, most of these protocols involve the direct contact between enzymes and supports, leading to enzyme-support interactions. Meanwhile, uniform deposition of enzymes on nano-scaled support is of problematic due to diffusion limit. In addition, fundamental studies are quite limited for the immobilized enzyme catalysis. For example, the support with hydrophobic surface has been claimed to be beneficial for promoting enzyme activity, but the opposite results were also observed^17^.

In this paper, we reported a general approach to prepare a novel immobilized enzyme by encapsulating the thin-layer polymer wrapped penicillin G amidase (PGA) into cage-like chambers of hierarchically mesoporous silica (HMS). As well known, PGA is widely used in industrial biotransformation, as a model system^18^. During water-medium hydrolysis of penicillin G potassium salt, such a catalyst (PNC-HMS) exhibited comparable activity to the naked PGA, and could be easily recycled and used repetitively for more than 5 times. The strong durability could be attributed to the cooperative effects from both the thin-layer polymer and the outer silica shell, which protected enzyme from leaching and structural deformation caused by heating and solvent corrosion. Besides, the thin-layer polymer wrapped glucose oxidase (GOD NCs) and horseradish peroxidase (HRP NCs) were co-encapsulated into HMS chambers, which acted as a bifunctional catalyst (GNC-HNC-HMS) in “one-pot” cascade reactions with high activity. Meanwhile, PNC-HMS catalyst displayed a low cellular cytotoxicity and GNC-HNC-HMS catalyst exhibited the similar catalytic activity for in vitro cell medium with that of the reaction in tube, showing good potential in industrial applications.

## Results

**Figure 1:**
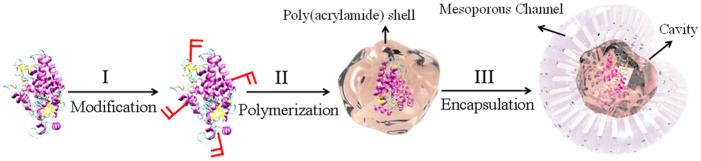
Schematic illustration of forming bioactive mesoporous composite by (I) covalently attaching polymerizable groups on an enzyme molecule by acrylation, (II) wrapping the modified protein molecule (core) with a poly(acrylamide) shell via in-situ polymerization, and (III) encapsulating enzyme nanocapsules inside the cavities of hierarchically mesoporous silica through physical adsorption.

Figure 1 illustrated the synthesis procedure of PNC-HMS catalyst. We firstly attached the polymerizable groups to PGA surface by reacting its resident lysine groups with N-acryloxysuccinimide (Step I). Subsequent in-situ polymerization in the presence of acrylamide (monomer), N, N′-methylene bisacrylamide (cross-linker), and N, N, N′, N′-tetramethylethylenediamine/ammonium persulfate (initiator) wrapped PGA with a thin poly(acrylamide) nanogel skin to generate PGA nanocapsules, named as PGA NCs (Step II). After gel filtration to remove unreacted proteins, monomers and initiators, PGA NCs could be easily encapsulated into mesoporous cavities to form PGA-HMS by putting the as-made hierarchically mesoporous silica in the PGA NCs aqueous buffer (Step III). For comparison, native penicillin G amidase without a polymeric nanogel skin was also encapsulated in the hierarchically mesoporous silica using the same protocol, named as PGA-HMS. Based on the enzyme amounts in solution before and after the immobilization measured with the Bradford method, the PGA loadings in PNC-HMS and PGA-HMS were determined as 2.5 wt% and 2.7 wt%, respectively. Moreover, we calculated the encapsulation efficiency of PNC-HMS sample by comparing the amount of PGA NCs encapsulated in the HMS support with the total nanocapsules used in the catalyst preparation. The enzyme encapsulation efficiency of PNC-HMS sample was determined as 7.8%. A subsequent wash with KCl aqueous solution did not obtain the additional PGA NCs in the washing solution, suggesting that PGA nanocapsules were not weakly adsorbed to the surface of the HMS support. For comparison, we also calculated the encapsulation efficiency of PGA-HMS sample by using native PGA enzyme. It obtained 8.5% encapsulation efficiency owing to its smaller size. To investigate the release kinetics of PNC-HMS and PGA-HMS samples, 50 mg catalyst was soaked in 100 mL 100 mM phosphate buffer and oscillated at 25°C for 24 h. The solution was sampled at given time intervals and the content of the left PGA enzyme in the solution was determined by the Bradford assay. As shown in Figure S1, the enzyme release in PNC-HMS sample was negligible (<10%) while PGA-HMS sample had a serious enzyme leaching with 40% in the same condition. This phenomenon could be explained by the large size of PGA nanocapsules in the PNC-HMS sample and the strong interaction between its hydrophilic polymeric shell of PGA NCs and the HMS support, which effectively inhibited the enzyme leaching.

**Figure 2:**
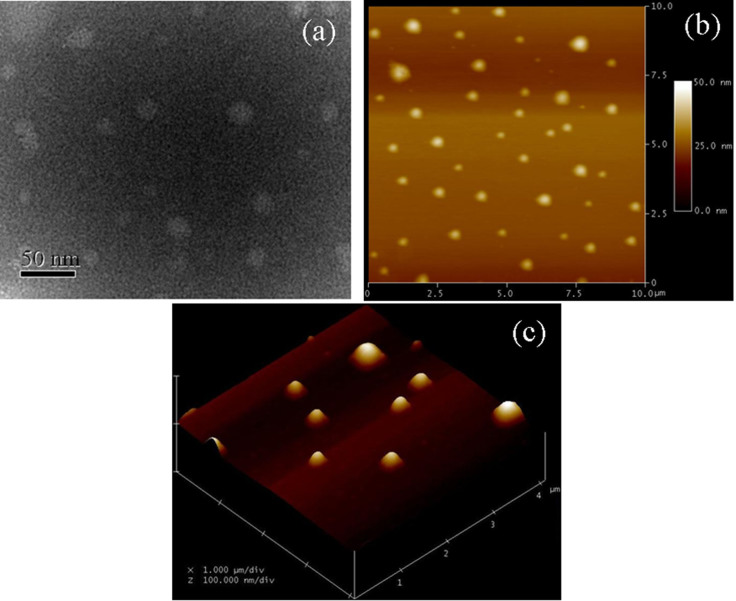
TEM (a) and AFM (b–c) images of PGA NCs sample.

**Figure 3:**
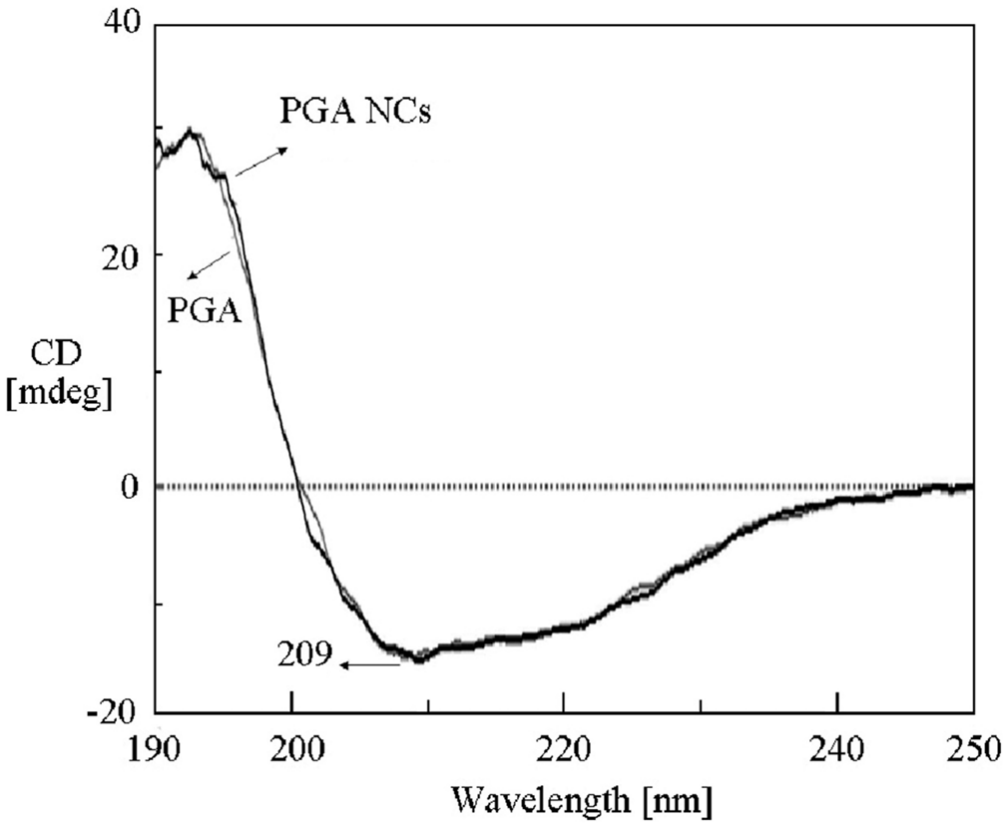
CD spectra of naked PGA enzyme and PGA NCs samples.

We firstly analyzed the size, shape and composition of PGA NCs. TEM picture (Figure 2a) of the negatively stained PGA NCs revealed a nearly spherical shape with the diameter of 20–30 nm. A dynamic light scattering (DLS) experiment revealed the average diameter of PGA nanocapsules were about 25 nm with a relatively low polydispersity value of 1.37 (Figure S2). 2D AFM image (Figure 2b) of PGA NCs on mica displayed the circular objects with a similar average diameter to that of TEM analysis. Meanwhile, AFM height measurement (Figure 2c) showed a typical height of about 25 nm, further confirming the nanoscale spherical structure of PGA NCs. Since the size of a free PGA molecule is 7.0 × 5.0 × 5.9 nm^3^^19^, the average shell thickness of PGA NCs was less than 10 nm. CD spectrum (Figure 3) of PGA NCs exhibited almost the same valley at 209 nm in comparison with native PGA molecules, indicating our in-situ polymerization process had no remarkable effect on compromising the structure of PGA molecules^20^. FTIR spectrum of the lyophilized PGA NCs sample (Figure S3) showed the characteristic absorptions of poly(acrylamide) at 1398, 1457 and 1644 cm^−1^, revealing the successful coating of a poly(acrylaimde) nanogel skin on the PGA molecule surface^21^. Moreover, the FTIR spectrum displayed an stronger absorbance at 3450 cm^−1^ corresponding to the absorbed water compared to native PGA molecule. It stated that PGA NCs could entrap significant amount of water, which was beneficial to retain the three dimensional conformation of PGA molecule^22^. The above results demonstrated unambiguously that PGA NCs have been successfully synthesized by using our aqueous in-situ polymerization approach.

**Figure 4:**
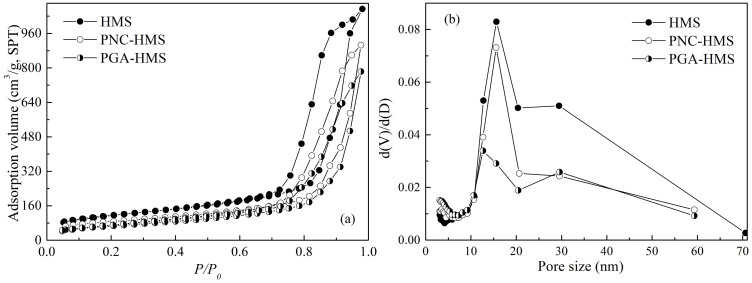
(a) N_2_ adsorption-desorption isotherms and (b) pore size distribution curves of HMS, PNC-HMS and PGA-HMS samples.

**Figure 5:**
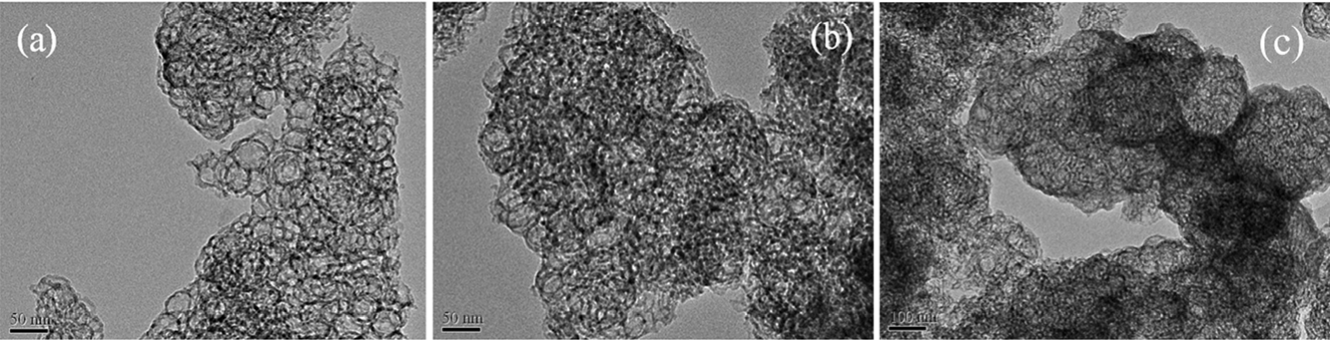
TEM images of (a) HMS, (b) PNC-HMS and (c) PGA-HMS samples.

The prefabricated hierarchically mesoporous silica was then employed to prepare PGA NCs-contained bioactive nanocomposite (PNC-HMS). Figure 4a revealed that the HMS support displayed the typical type IV N_2_ adsorption-desorption isotherm with a H_1_ hysteresis loop indicative of mesoporous structure^23^. Pore size distribution curve (Figure 4b) confirmed that it contained two distinct types of pores centered at 30 and 15 nm. High-resolution TEM image of the HMS support (Figure 5a) exhibited a uniform mesocellular cage-like structure. It also revealed that the larger 30 nm cellular pores in the HMS sample were well-mixed with the smaller 15 nm ordered mesopores^24^. Both PNC-HMS and PGA-HMS samples showed similar N_2_ sorption isotherms to that of HMS support, confirming that they could maintain hierarchically mesoporous structure. The surface area and the total pore volume decreased for PNC-HMS and PGA-HMS samples (Table 1), indicating the successful incorporation of PGA NCs or PGA in the HMS matrix. Interestingly, pore size distribution analysis (Figure 4b) revealed that the pore size corresponding to the mesoporous structure (15 nm) in the PNC-HMS sample remained almost unchanged but the pore size corresponding to the cavities (30 nm) decreased remarkably. On contrast, PGA-HMS sample exhibited an abrupt decrease in the pore size corresponding to mesopore (15 nm), but the pore size corresponding to the cavity (30 nm) remained almost unchanged. The reproducibility of these curves in Figure 4 was checked by repeating each result at least three times and the results revealed that N_2_ sorption isotherms and pore size distributions of HMS, PNC-HMS and PGA-HMS samples were reproducible. In addition, HRTEM image (Figure 5b) revealed that PNC-HMS sample displayed almost the same HRTEM image to that of HMS material, but the cavities were partially filled by PGA NCs through the open mesoporous channels. This could be explained by the inaccessibility of PGA NCs to the mesoporous channels due to their large particle size (20 ~ 30 nm). Encapsulation of PGA NCs in the HMS support could be further confirmed by fluorescence micrograph using fluorescein isothiocyanate (FTIC) modified PGA NCs since the labeled PNC-HMS showed a typical green fluorescence image (Figure S4). Nevertheless, the empty cavities in the PGA-HMS sample could be clearly observed (Figure 4c), demonstrating most PGA molecules might occupy mesoporous channels since native PGA molecule displayed much smaller size than mesopore size. Thus, we inferred from these observations that PGA NCs should exist in the large cavities of HMS material, while the remaining mesopores allowed the substances to access the active sites of PGA NCs^25^.

## Discussion

**Figure 6:**
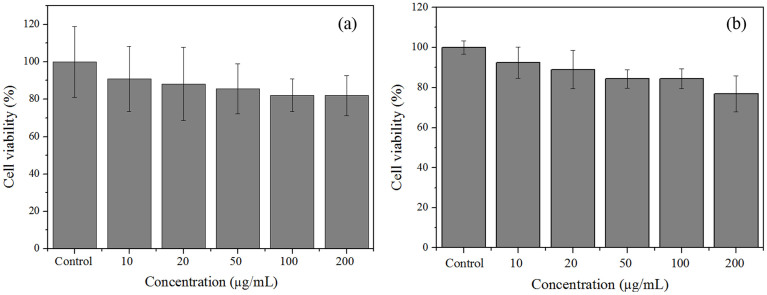
HeLa cell viability after incubation with PGA NCs (a) and PGA-HMS (b) samples for 24 h at different concentrations.

The hydrolysis of penicillin G potassium salt in water was used as a probe to evaluate the biological activity of PGA enzyme in different states. Besides the apparent activity (R_apperant_), the kinetic constant K_M_ and k_cat_ were also determined by Michaelis-Menten equation. As shown in Table 1, PGA NCs displayed almost the same R_apperant_ (45 U/mg), K_M_ (20 mM) and k_cat_ (3.2 min^−1^) as those of native PGA (48 U/mg, 20 mM and 3.2 min^−1^), indicating it retained most of its original enzymatic activity. This could be mainly ascribed to the extremely thin-layer polymer skin, which was highly permeable and thus allowed the convenient transport of substrates into PGA active sites. In comparison with PGA NCs, PNC-HMS sample showed only a little decrease in R_apperant_ (39 U/mg), K_M_ (18 mM) and k_cat_ (2.8 min^−1^). However, PGA-HMS sample displayed an abrupt decrease in R_apperant_ (32 U/mg), K_M_ (15 mM) and k_cat_ (2.1 min^−1^). Since PGA-HMS and PNC-HMS samples displayed the similar mesoporous structure, the difference in their activity could be attributed to different chemical microenvironment of the PGA existed. In the PGA-HMS sample, PGA NCs has a polyacrylamide nanogel skin, which provided a hydrophilic protecting layer around PGA molecule and hence avoided the direct contact with the HMS support. The direct contact may cause the structural deformation of PGA molecule since the isoelectric point of PGA is around 6.5, making it negative at pH 7.8 in the adsorption condition. The silanol groups of HMS support were also negative at this pH. As a result, the electrostatic repulsion generated from the enzyme-support interaction was unfavorable to retain PGA original conformation^26^. Accordingly, the deformation of PGA molecule might cause the reduced enzyme flexibility for matching the structure of reactant molecules^17^, leading to the reduce in activity for PGA-HMS catalyst. More importantly, the thin-layer poly(acrylaimde) layer covering the PGA molecule in the PNC-HMS catalyst showed strong ability for water adsorption, which was also beneficial to retaining PGA molecular conformation^15^. Another reason was that the surrounded unoccupied mesoporous channels in the PNC-HMS sample facilitated the reactant molecules to pass through silica shell into the cavities, resulting in the decreased diffusion resistance^27^. Therefore, we concluded that the high bioactivity of PNC-HMS catalyst was generated from the unique combinational structure, which could prevent the structural deformation of PGA molecules and simultaneously diminish mass transfer resistance. In addition, we measured the cell viability after incubating HeLa cells with PGA NCs and PNC-HMS samples. As presented in Figure 6, the cell viability of PGA NCs and PGA-HMS remained above 75% even at the highest concentration of 200 μg/mL after the incubation for 24 h, indicating the low cellular cytotoxicity^20^.

To further confirm the advantage of this soft-hard cooperative approach, we extended to develop multifunctional bioactive nanocomposite for one-pot tandem reaction^28^. According to the similar protocol, we synthesized the polymer nanogel wrapping glucose oxidase nanocapsules (GOD NCs) and horseradish peroxidase nanocapsules (HRP NCs) and then co-encapsulated them into HMS cavities, which was denoted as GNC-HNC-HMS. For comparison, naked GOD and HRP were also co- deposited onto HMS support, named as GOD-HRP-HMS. Besides, polymer nanogel wrapped GOD NCs or HRP NCs were also individually deposited onto HMS matrix, denoted as GNC-HMS and HNC-HMS, respectively. The successful encapsulation of two bioactive nanocapusles were confirmed by fluorescence images using the prefabricated fluorescein isothiocyanate (FTIC) labelled GOD NCs and Rhodamine B labelled HRP NCs, which displayed green and red colors at the same time (Figure S5).

A tandem reaction consisting of GOD-catalyzed glucose conversion to lactone to release H_2_O_2_ and the subsequent HRP-catalyzed ABTS conversion to ABTS^+^ was used as a probe to evaluate the catalytic performance of GNC-HNC-HMS catalyst^29^. As shown in Table 2, it showed the similar specific activity (165 U/mg), K_M_ (119 mM) and k_cat_ (4.8 min^−1^) to homogeneous enzymes system containing native GOD and HRP (162 U/mg, 112 mM and 5.0 min^−1^). This result demonstrated that the original activities of both GOD and HRP did not significantly affected by wrapping with thin-layer polymer and encapsulating with HMS. Interestingly, it exhibited much higher activity than that of GOD-HRP-HMS sample, which furthermore confirmed that the use of enzyme nanocapsules instead of native enzymes played a key role in preserving the enzyme activities after being encapsulated into HMS material. It was also found that the GNC-HNC-HMS sample displayed superior catalytic activity than either the mixture of homogeneous GOD solution and HRP NCs encapsulated in the HMS cavities (HNC-HMS) or the mixture of homogeneous HRP solution and GOD NCs encapsulated in the HMS cavities (GNC-HMS), showing a cooperative effect between GOD NCs and HRP NCs. However, no cooperative effect between naked GOD and HRP occurred in the mixture of neither GOD and HNC-HMS nor HRP and GNC-HMS since neither native GOD nor native HRP molecule was hard to pass through hydrophilic silica shell to meet the other enzyme in the HMS cavities due to the strong surface hydrophilicity and large molecular size of GOD or HRP. Moreover, we extended this catalytic system to in vitro HeLa cell medium. As shown in Table 2, the results revealed that the enzymatic activity in cell displayed almost the same activity with that in the tube. Meanwhile, the treated cell viability remained around 60%, indicating the slight damage of GNC-HNC-HMS catalytic process for cells.

**Figure 7:**
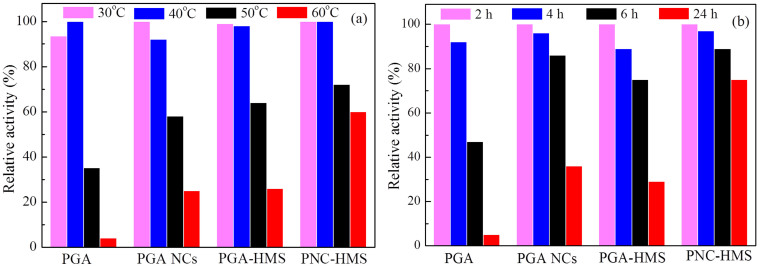
(a) Thermal stability test of different enzymes after treating 2 h at different temperatures and (b) Stability test of different enzymes in buffer solution containing 10% (v/v) butyl acetate and 90% (v/v) 100 mM phosphate (pH = 7) at 37°C for 24 h. The results were the average of at least three independent stabiltiy test.

**Figure 8:**
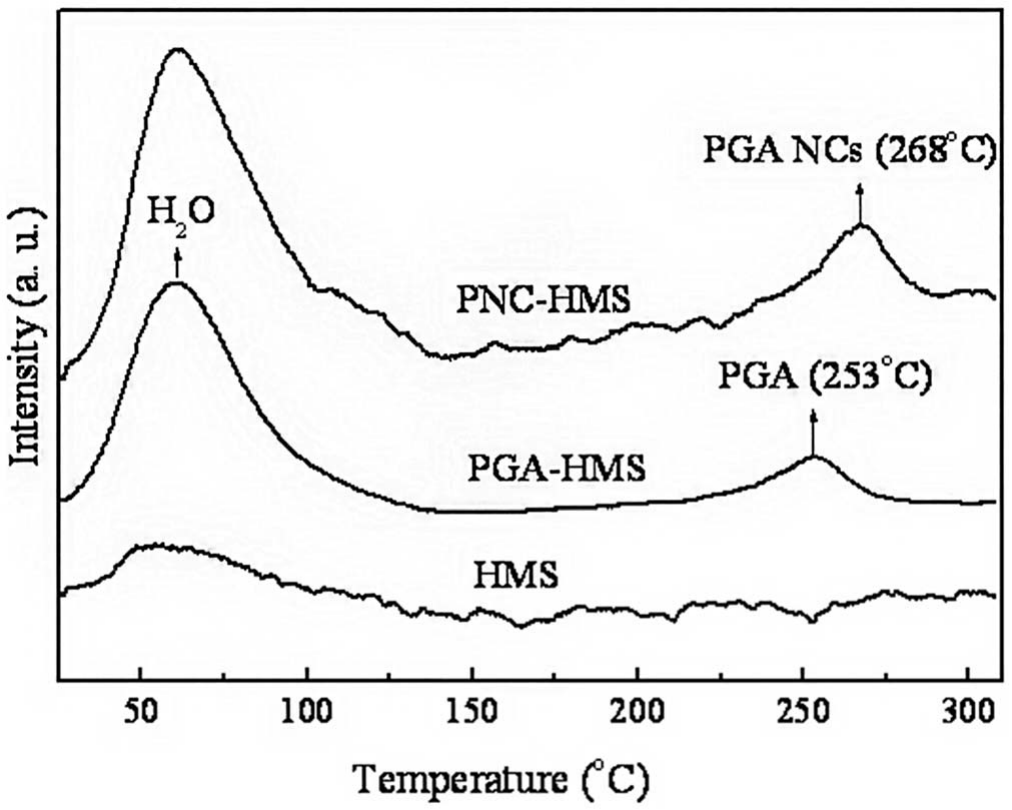
TPD profiles of HMS, PGA-HMS and PNC-HMS samples.

The stability of enzyme at high temperature and in organic solvents is critical to its industrial applications^30^. As a model system, we compared the stability of native PGA, PGA NCs, PGA-HMS and PNC-HMS samples against heating and organic co-solvent corrosion. The results (Figure 7) revealed that PGA NCs displayed better stability than free PGA since the plentiful water molecule in the polymeric skin could help enzyme to retain its structure and function. More interestingly, incorporation of PGA NCs into hierarchically mesoporous silica further enhanced enzyme stability. It could be clearly found that PNC-HMS composite had the optimal stability both at high temperature and in organic solvents soaking treatment. For example, it still retained 60% ± 3.5% and 75% ± 2.7% of the original activity even at 60°C for 2.0 h and in organic-buffer mixed solvent for 24 h, respectively. It was much higher than those of native PGA (4% ± 0.2% and 5% ± 0.1%), PGA NCs (25% ± 1.5% and 36% ± 1.0%) and PGA-HMS (26% ± 2.4% and 29% ± 2.0%). To investigate the reason for the excellent stability of PNC-HMS sample, we used temperature-programmed desorption (TPD) technology to analyze the interaction between enzyme and HMS material (Figure 8). HMS sample displayed only one desorption peak corresponding to water at 62°C. However, PGA-HMS and PNC-HMS samples showed an additional peak corresponding to enzyme desorption at higher temperature. Noted that the desorption temperature of PNC-HMS sample had a remarkable increase of about 15°C compared to that of PGA-HMS sample. This higher desorption temperature of PNC-HMS sample indicated that the encapsulated PGA NCs existed more strongly in the HMS material. As a consequence, it was expected to increase its stability at high temperature and in organic co-solvents^31^. Base on these results, we inferred that PNC-HMS composite constructed unique dual protections derived from the soft polymer nanogel and hard mesoporous silica shell. This combinational soft/hard structure could retard the solvent corrosion by inhibiting the PGA from direct attack of solvent molecules. Meanwhile, they could also diminish the deformation of PGA three dimension structures at high temperature due to the retard of heat transfer and heat sink^32^.

**Figure 9:**
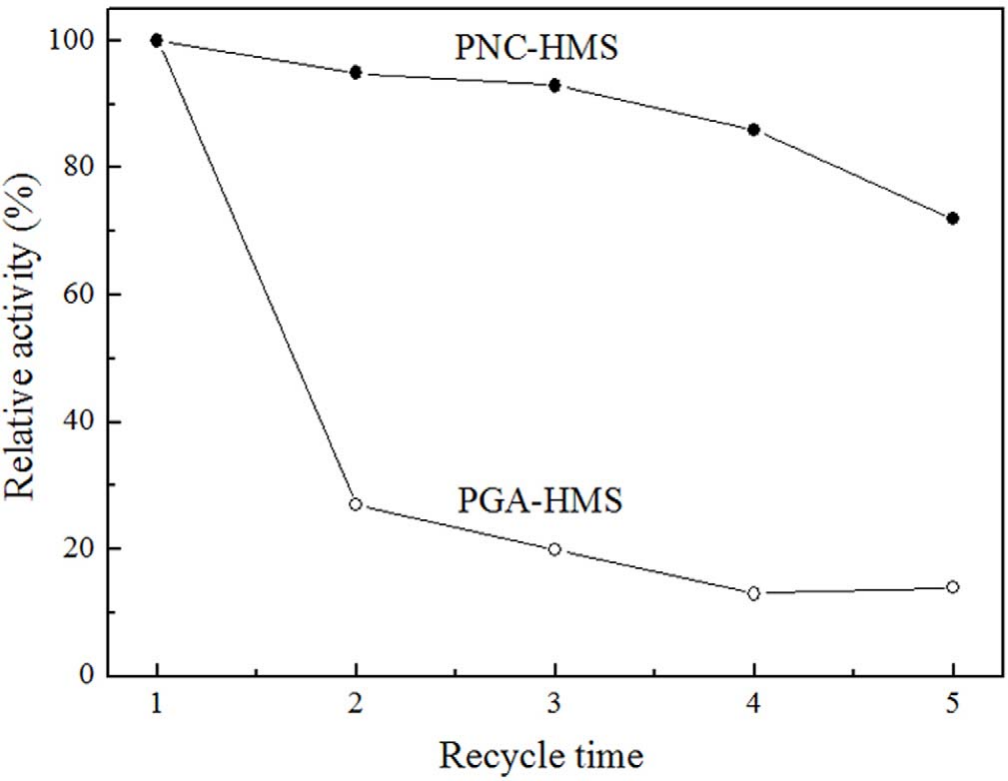
Recycling tests of PNC-HMS and PGA-HMS catalysts in the hydrolysis reaction of penicillin G potassium salt. The results were the average of at least three independent recyclability test.

Furthermore, we evaluated the recyclability of PNC-HMS composite by checking its catalytic activities at different cycles in water-medium hydrolysis of penicillin G potassium salt. The PNC-HMS sample displayed no significant decrease in activity in the second run and only less than 30% ± 2.0% decrease in activity after being used repetitively for 5 times. However, the PGA-HMS showed rapid decrease in activity and about 70% ± 4.5% activity was lost in the second run of reactions (Figure 9). These results demonstrated the excellent durability of PNC-HMS nanocomposite. The fast decay of PGA-HMS sample originated from the leaching of PGA molecule, which was detected in the supernatant. No significant PGA NCs leaching was observed for over five cycles, and it still maintained hierarchically mesoporous structure after catalytic cycles as evidenced by TEM image (Figure S6). Meanwhile, the pore surface of mesoporous silica support and the polymeric nanogel were hydrophilic, making their contact more favorable and compact. Moreover, PNC-HMS sample retained 86% its apparent activity after being stored at 25°C for 30 days, showing great storage stability; however, PGA-HMS sample only remained 47% activity in the same condition (Figure S7). We reasoned that the excellent stability in the reuse and storage of PNC-HMS was because that PGA NCs were trapped in the mesoporous cavities, preventing their escape from the support.

In summary, we have demonstrated the successful soft-hard cooperative approach for the synthesis of novel bioactive mesoporous composite through encapsulating bioactive nanocapsules into hierarchically mesoporous silica. The resulting nanocomposites exhibited high activity and excellent stability, which could be attributed to the synergic effect generated from the soft polymer nanaogel skin and hard mesoporous silica shell. This unique structure effectively provided a favorable heterogeneous condition for biomolecules and simultaneously decreased the undesired enzyme-support interactions and the mass transfer limitation. This design concept can be generalized to fabricate a large variety of bioactive mesoporous composites with improved catalytic performances for industrial applications.

## Methods

### Preparation of PGA nanocapsules (PGA NCs)

10 mL 20 mg/mL PGA was dissolved in 90 mL 100 mM phosphate buffer solution with pH of 8.0, followed by adding dropwise 4.0 mL DMSO solution containing 20 mg N-acryloxysuccinimide and reacting at 20°C for 1.0 h. The acrylated PGA was purified by gel filtration on Sephadex G-25 column. 20 mL solution containing 1.0 mg/mL acrylated PGA was prepared in a vial and purged with nitrogen. The radical polymerization from the surface of the acrylated PGA was started by adding 3.0 mg of ammonium persulfate dissolved in 30 μL deoxygenated and deionized water solution and 3.0 μL of N, N, N′, N′-tetramethylethylenediamine. Then, the amount of monomers, acrylamide and N, N′-methylene bisacrylamide (mol/mol = 5/1), dissolved in 2.0 mL deoxygenated and deionized water were evenly added with monomer/PGA molar ratio of 600. After reaction at 20°C for 2.0 h under nitrogen atmosphere, gel filtration with Sephadex G-75 was applied to remove the unreacted monomers and initiators, leading to PGA NCs.

### Preparation of HRP nanocapsules (HRP NCs)

100 mg of HRP and 10 mg of 4-dimethylaminoantipyrine (stabilizer of HRP) were firstly dissolved in 38 mL of pH 9.3, 100 mM boric acid buffer. Then 40 mg of N-acryloxysuccinimide dissolved in 2 mL of DMSO was slowly added and the reaction was carried out for 2.0 h at 20°C. The acrylated HRP was purified by gel filtration on Sephadex G-25 column. A 3.5 mL solution containing a specific weight of acrylated HRP with specific amount of 4-dimethylaminoantipyrine was prepared in a vial and purged with nitrogen. The radical polymerization from the surface of the acrylated HRP was started by adding 3.0 mg of ammonium persulfate dissolved in 30 μL of deoxygenated and deionized water and 3.0 μL of N, N, N′, N′-tetramethylethylenediamine into the test tube. Then specific amount of monomers, acrylamide and N, N′-methylene bisacrylamide (mol/mol = 10/1), dissolved in 0.50 mL deoxygenated and deionized water was evenly added to the test tube in 1.0 h. The monomer/HRP molar ratio of 800 was setted. The reaction proceeded for another 1.0 h at nitrogen atmosphere. Finally, gel filtration with Sephadex G-75 was applied to remove the unreacted monomers and initiators.

### Preparation of GOD nanocapsules (GOD NCs)

The protocol of synthesize GOD nanocapsules was similar to that of HRP NCs. The enzyme content was determined by bicinchoninic acid (BCA) colorimetric protein assay.

### Synthesis of Hierarchically Mesoporous Silica (HMS)

1.0 g of P123 and 0.42 mL of concentrated acetic acid were dissolved in 20 mL water. The mixing solution was heated to 60°C for 1.0 h and then 1.6 mL of sodium silicate was dropped into the solution with vigorous stirring. After reacting for 2.0 h, the solution was aged at 80°C for another 20 h, followed by the hydrothermal treatment at 100°C for 24 h. The surfactant was removed by calcination at 550°C, resulting in the final hierarchically mesoporous silica, named as HMS.

### Synthesis of PGA NCs encapsulated HMS (PNC-HMS)

0.25 g HMS was added into 10 mL 0.80 mg/mL PGA NCs buffer solution (pH 7.8, 100 mM). The solution was shaken at 30°C for 12 h at a rotating speed of 100 ppm. The solid product was filtered and washed with deionized water and buffer, leading to PNC-HMS. For comparison, naked PGA enzyme was also encapsulated into HMS support using the same procedure, named as PGA-HMS.

### Synthesis of HRP NCs and GOD NCs co-encapsulated HMS (GNC-HNC-HMS)

The GNC-HNC-HMS was synthesized by co-encapsulating GOD NCs and HRP NCs into HMS chamber in the same way used for synthesizing PNC-HMS. Meanwhile, the GOD-HRP-HMS was also fabricated by co-encapsulating the naked GOD and HRP into the HMS chamber following the same procedure used for fabricating PGA-HMS.

### Characterization

FTIR spectra were recorded on a Nicolet Magna 550 spectrometer. Transmission electron microscopy (TEM) was performed using a JEOL 2011 electron microscope. During a run for PGA NCs, the sample was diluted in water to give concentrations of 0.5 mg/mL. Carbon-coated grid was prepared by adding a drop of enzyme nanocapsules solution, removing the excess, and applying 1%, pH 7.0 sodium phosphotungstate. The sample was then subjected to TEM measurement. AFM measurement of PGA NCs was performed with a Veeco Multimode IIIa instrument operated in the tapping mode in air. A new mica surface was prepared by fresh cleaving and then exposed to enzyme solution of 3.0 μM for 30 s. CD spectra (190–250 nm) were performed with a Jasco-810 instrument in a quartz cuvette with a 1.0 mm path length. DLS was performed using a DynaPro-801 dynamic light scattering instrument (Protein Solutions, Co.). Data were collected and analyzed using the AutoPro data software for the DynaPro-801 instrument (Protein Solutions, Co.). For the measurements, the enzyme concentration was adjusted to 5.0 μM. Each sample was scanned three times at 20 nm/min with a step length of 0.5 nm. N_2_ sorption isotherms were obtained on a Nova 4000 analyzer at 77 K. Specific surface areas (S_BET_) and average pore diameter (D_P_) are calculated by using BET and BJH models, respectively based on the adsorption branches. Temperature-programmed desorption curve was conducted on a Micromeritics Autochem II 2920 instrument in Ar flow at a ramping rate of 5.0 K/min.

### PGA activity assay

For testing the activity of PGA enzyme, the buffer solution containing 2.2 mg PGA in naked PGA, PGA NCs, PNC-HMS, or PGA-HMS and 10 mL 100 mM phosphate buffer (pH = 7.8) were mixed and kept at 37°C in the thermostatic bath, and then 10 mL 4.0% (w/v) aqueous solution of penicillin G potassium salt was added. The solution was automatically titrated with 0.10 mol/L NaOH solution to maintain pH = 7.8. The volume of NaOH consumed during the first 5.0 min was measured. The specific activity of free and immobilized PGA was calculated as follows: A (U/mg) = VNaOH × CNaOH × 10^3^/m × t, where m and t referred to PGA content and reaction time, respectively.

### GOD-HRP activity assay

For testing the activity of cooperative GOD and HRP enzymes: 1.0 mL phosphate buffer solution containing (a) naked GOD and naked HPR, (b) GNC-HNC-HMS, (c) GOD-HRP-HMS, (d) naked GOD and HNC-HMS or (e) naked HRP and GNC-HMS with the same protein loading and GOD/HRP ratio (1/1) was transferred into a cuvette, followed by adding 100 μL 1.8 mM ABTS solution in phosphate buffer and 1.0 mL 0.1 mg/mL β-D-glucose. The catalytic activity was determined according to product analysis on a UV-Vis spectrometer at the characteristic wavelength of 420 nm.

### GOD-HRP activity assay in cells

Firstly, we plated HeLa cells in a 96-well plate (5000 cells/well) for 1 day prior to the exposure of catalyst and cultured the cells with normal medium. 1.0 mg GNC-HNC-HMS catalyst was added into cell medium and subsequently introduced ABTS buffer solution to cells to reach a final concentration of 0.5 mM. After that, 0.1 mL 0.2 mg/mL β-D-glucose solution was added into the treated cells. The catalytic activity was determined in the first 15 min. After the activity test, the cells were rinsed with normal culture medium and placed back to the incubator for 4.0 h for measure the cell viability with MTT assay.

### MTT assay

In vitro cytotoxicity of PGA NCs and PGA-HMS samples were evaluated by performing methyl thiazolyl tetrazolium (MTT) assay of HeLa cells. The Cells were seeded into a 96-well cell culture plate at a density of 5 × 10^4^/well and cultured in RPMI-1640 with 10% FBS and 1% penicillin-streptomycin at 37°C and 5% CO_2_ for 24 h. The next day, the cells were incubated with PGA NCs or PGA-HMS samples with different concentrations of 0, 10, 20, 50, 100 and 200 μg/mL diluted in RPMI-1640 for another 1 24 h at 37°C under 5% CO_2_. Thereafter, MTT (10 μL, 5000 μg/mL) was added to each well and the plate was incubated for an additional 4.0 h at 37°C. After removal of the medium, the purple formazan product was dissolved with DMSO for 15 min. Finally, the optical absorption of formazan at 490 nm was measured by an enzyme-linked immunosorbent assay reader, with background subtraction at 690 nm was measured by microplate reader (Multiskan MK3, USA).

### Determination of storage stability and reusability

Free PGA solution containing 0.10 mg/mL enzyme was kept at 25°C and residual activities of these samples were measured periodically for one month. The same type of measurements was carried out by using immobilized samples incubated as dried solid forms. In order to determine the catalyst reusability, the PGA-HMS or PNC-HMS nanocomposite was allowed to settle down after each run of reactions and the clear supernatant liquid was decanted slowly. The residual solid sample was re-used with fresh charge of buffer and reactant for subsequent recycle runs under same reaction conditions.

## Author Contributions

H.L. and F.Z. designed the experimental scheme and analyzed the experimental data. F.Z. and M.W. did the most of the sample preparation and characterizations. F.Z. and H.X. wrote and revised the manuscript. C.L., H.J. and J.S. helped with the experiment.

## Supplementary Material

Supplementary Information supporting information

## Figures and Tables

**Table 1:** Structural parameters and catalytic performances[a]

Sample	Loading (wt.%)	S_BET_ (cm^2^/g)	V_p_ (cm^3^/g)	R_apprent_ (U/mg)	K_M_ (mM)	k_cat_ (min^−1^)
HMS	/	351	1.5	/	/	/
PGA	/	/	/	48	22 ± 3.5	3.7 ± 0.3
PGA NCs	/	/	/	45	20 ± 2.1	3.2 ± 0.2
PNC-HMS	2.5	308	1.4	39	18 ± 1.5	2.8 ± 0.3
PGA-HMS	2.7	240	1.2	32	15 ± 2.5	2.1 ± 0.2

^[a]^Reactions were carried out at 37°C in 100 mM phosphate buffer (pH = 7.8) using 10 mL 4.0% (w/v) aqueous solution of penicillin G potassium salt and 2.2 mg PGA enzyme.

**Table 2:** Catalytic properties of different multifunctional enzyme systems[a]

Sample	R_apprent_ (U/mg)	K_M_ (mM)	k_cat_ (min^−1^)
GOD + HRP	165	112 ± 8.9	5.0 ± 0.2
GNC-HNC-HMS	162	119 ± 8.1	4.8 ± 0.3
GOD-HRP-HMS	136	136 ± 9.8	3.3 ± 0.1
GOD + HNC-HMS	145	127 ± 8.5	3.8 ± 0.2
HRP + GNC-HMS	149	131 ± 9.1	3.6 ± 0.1
GNC-HNC-HMS[b]	151	132 ± 9.3	3.7 ± 0.2

^[a]^Reactions were carried out at 25°C in 100 mM phosphate buffer (pH = 4.5) using 1.0 mL 1.0 mg/mL β-D-glucose solution, 100 μL 1.8 mM ABTS, 0.1 mg GOD enzyme and 0.1 mg HRP enzyme.

^[b]^Rection in in a 96-well plate (5000 HeLa cells/well), 0.1 mg GNC-HNC-HMS catalyst, 0.1 mL 0.2 mg/mL β-D-glucose solution, 10 μL 0.5 mM ABTS.
