# Supplementary material for: Thin-Layer Polymer Wrapped Enzymes Encapsulated in Hierarchically Mesoporous Silica with High Activity and Enhanced Stability
Source: Sci Rep. 2014 Mar 21;4:4421. doi: 10.1038/srep04421 (PMC3961738; doi:10.1038/srep04421)
Supplement: Supplementary Information — supporting information [file srep04421-s1.doc]

Supporting information for

Thin-Layer Polymer Wrapped Enzymes Encapsulated in Hierarchically Mesoporous Silica with High Activity and Enhanced Stability

Fang Zhang, Meitao Wang, Chao Liang, Huangyong Jiang, Jian Shen & Hexing Li

The Education Ministry Key Lab of Resource Chemistry and Shanghai Key Laboratory of Rare Earth Functional Materials, Shanghai Normal University, Shanghai 200234, China

*
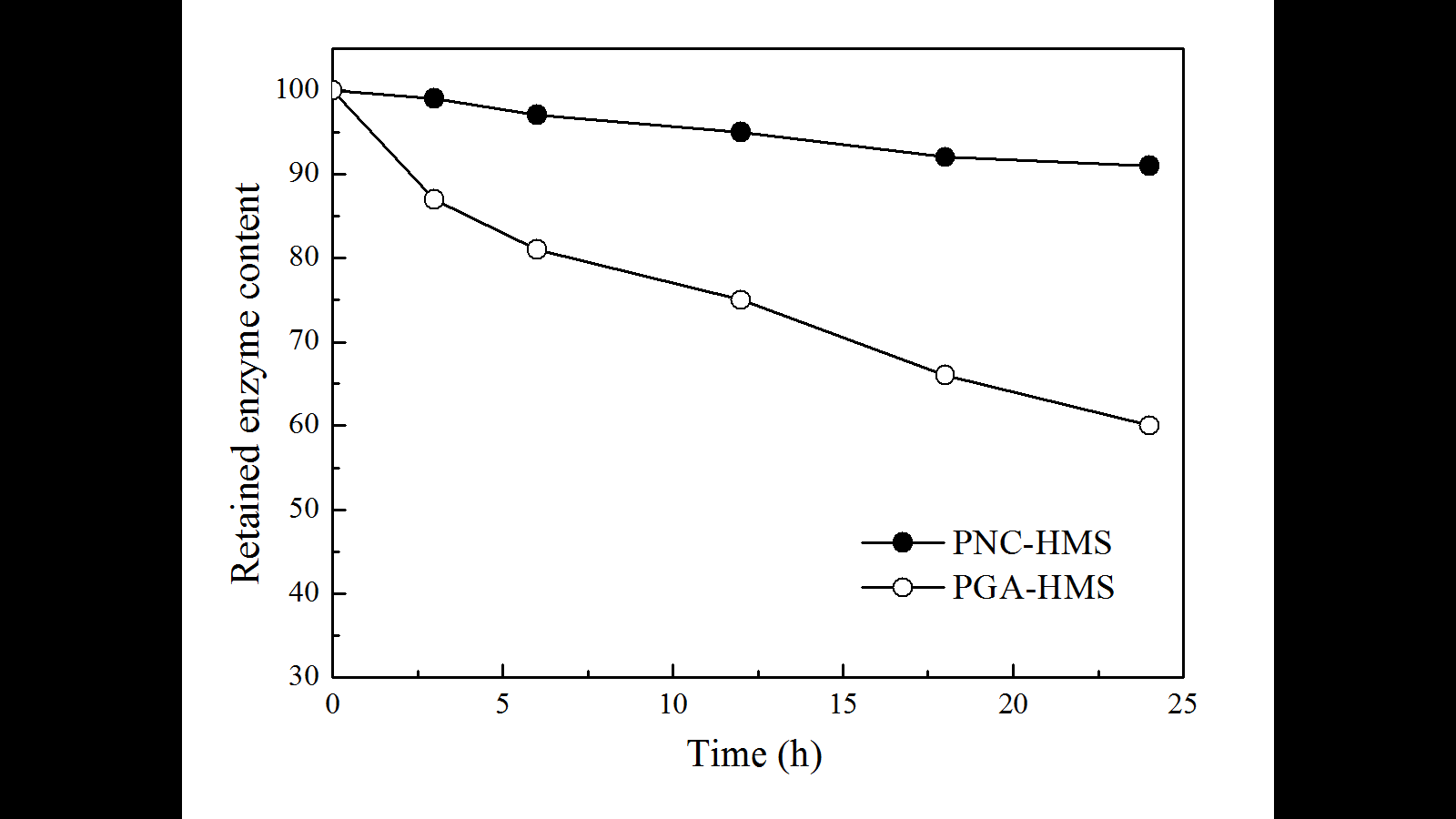
*

Figure S1. Release kinetics files over PNC-HMS and PGA-HMS samples in buffer solution.

*
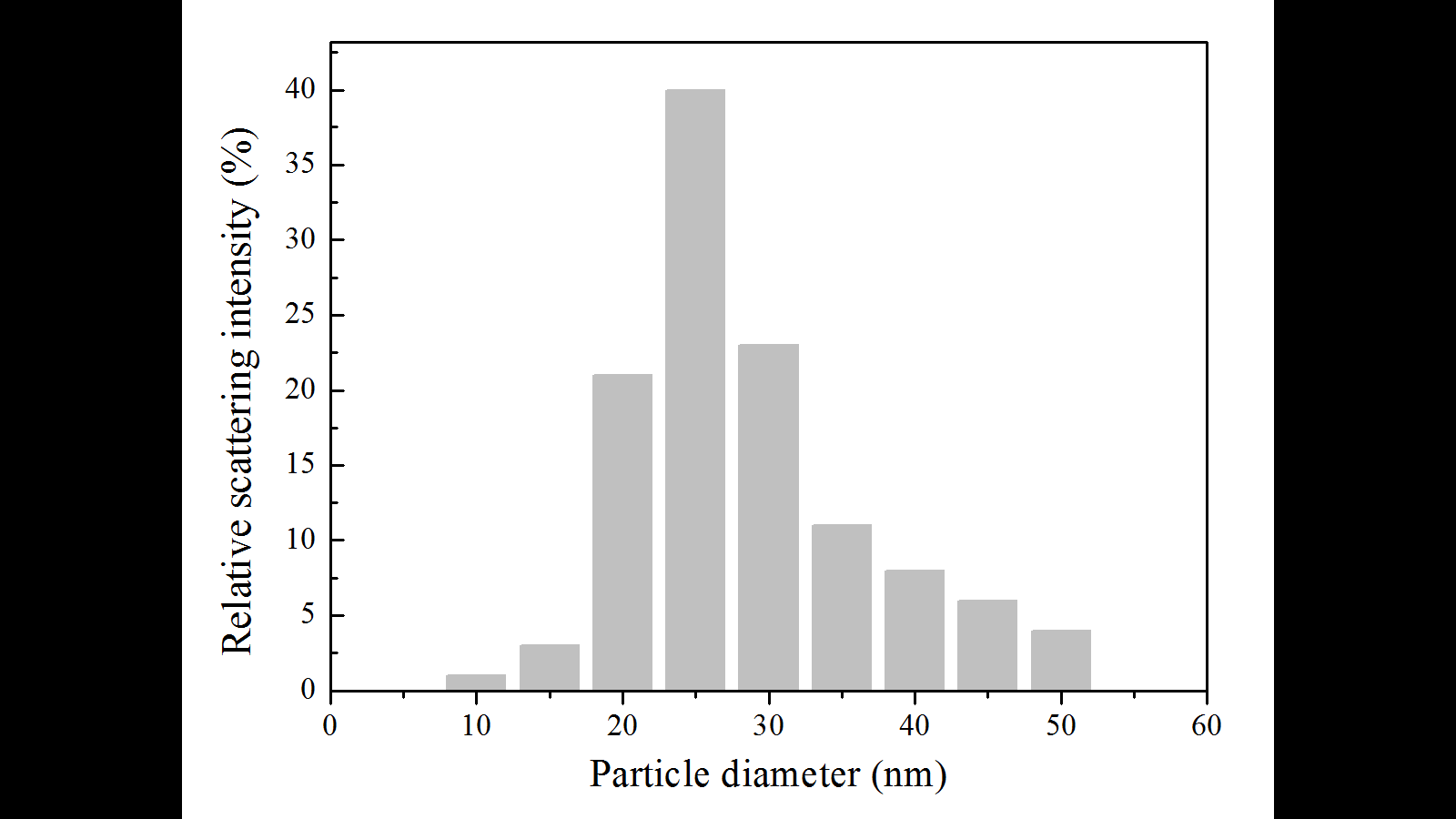
*

Figure S2. DLS size-distribution diagram of PGA nanocapsules.

Figure S3. FTIR spectra of naked PGA and PGA NCs samples.


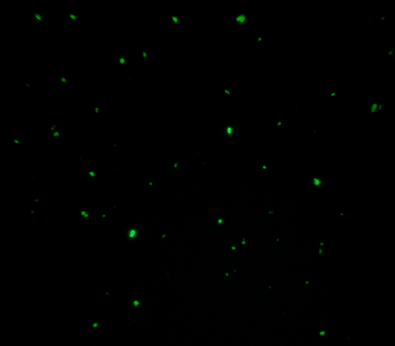


Figure S4. Fluoroscope image of the labeled PNC-HMS composite that prepared by using FITC labeled PGA NCs.


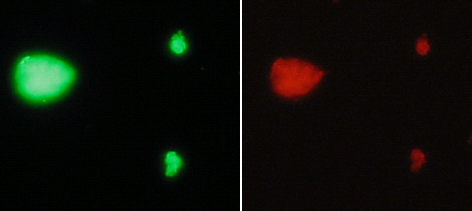


Figure S5. Fluoroscope images of GNC-HNC-HMS composite that prepared by using the prefabricated FITC labeled GOD NCs and Rhodamine B labeled HRP NCs.


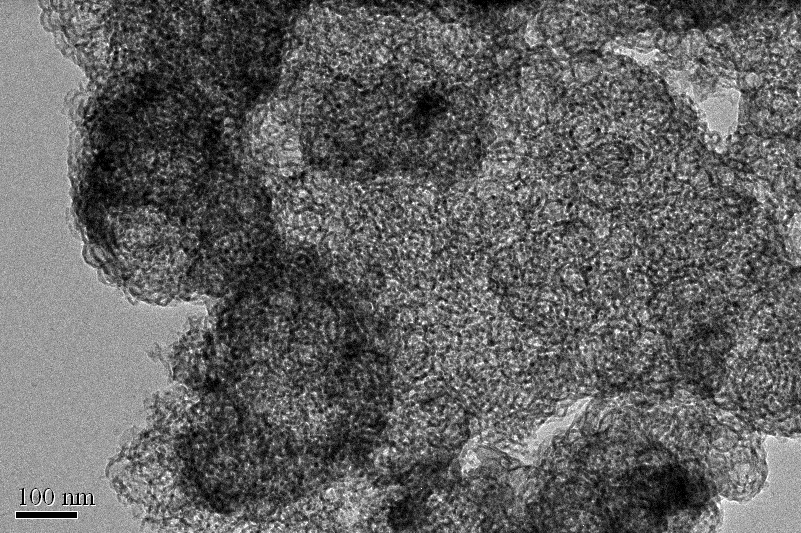


Figure S6. TEM image of PNC-HMS sample after being reused for 5 times.

Figure S7. Storage stability test of PGA-HMS and PNC-HMS samples in air at 25^o^C.
